# Groundwater Depths Affect Phosphorus and Potassium Resorption but Not Their Utilization in a Desert Phreatophyte in Its Hyper-Arid Environment

**DOI:** 10.3389/fpls.2021.665168

**Published:** 2021-06-07

**Authors:** Bo Zhang, Gangliang Tang, Hui Yin, Shenglong Zhao, Muhammad Shareef, Bo Liu, Xiaopeng Gao, Fanjiang Zeng

**Affiliations:** ^1^Xinjiang Key Laboratory of Desert Plant Roots Ecology and Vegetation Restoration, Xinjiang Institute of Ecology and Geography, Chinese Academy of Sciences, Urumqi, China; ^2^National Engineering Technology Research Center for Desert-Oasis Ecological Construction, Xinjiang Institute of Ecology and Geography, Chinese Academy of Sciences, Urumqi, China; ^3^Cele National Station of Observation and Research for Desert-Grassland Ecosystems, Cele, Xinjiang, China; ^4^Northwest Institute of Eco-Environment and Resources, Academy of Science, Lanzhou, China; ^5^College of Resources and Environment, Linyi University, Linyi, China; ^6^Department of Soil Science, University of Manitoba, Winnipeg, MB, Canada; ^7^State Key Laboratory of Desert and Oasis Ecology, Xinjiang Institute of Ecology and Geography, Chinese Academy of Sciences, Urumqi, China

**Keywords:** nutrient resorption, *Alhagi sparsifolia*, groundwater table, internal nutrient cycling, desert ecosystem

## Abstract

Nutrients are vital for plant subsistence and growth in nutrient-poor and arid ecosystems. The deep roots of phreatophytic plants are necessary to access groundwater, which is the major source of nutrients for phreatophytes in an arid desert ecosystem. However, the mechanisms through which changes in groundwater depth affect nutrient cycles of phreatophytic plants are still poorly understood. This study was performed to reveal the adaptive strategies involving the nutrient use efficiency (NUE) and nutrient resorption efficiency (NRE) of desert phreatophytes as affected by different groundwater depths. This work investigated the nitrogen (N), phosphorus (P), and potassium (K) concentrations in leaf, stem, and assimilating branch, as well as the NUE and NRE of the phreatophytic *Alhagi sparsifolia.* The plant was grown at groundwater depths of 2.5, 4.5, and 11.0 m during 2015 and 2016 in a desert-oasis transition ecotone at the southern rim of the Taklimakan Desert in northwestern China. Results show that the leaf, stem, and assimilating branch P concentrations of *A. sparsifolia* at 4.5 m groundwater depth were significantly lower than those at 2.5 and 11.0 m groundwater depths. The K concentrations in different tissues of *A. sparsifolia* at 4.5 m groundwater depth were significantly higher than those at 2.5 and 11.0 m groundwater depths. Conversely, the NRE of P in *A. sparsifolia* was the highest among the three groundwater depths, while that of K in *A. sparsifolia* was the lowest among the three groundwater depths in 2015 and 2016. The N concentration and NUE of N, P, and K in *A. sparsifolia*, however, were not influenced by groundwater depth. Further analyses using structural equation models showed that groundwater depth had significant effects on the P and K resorption of *A. sparsifolia* by changing soil P and senescent leaf K concentrations. Overall, our results suggest groundwater depths affect P and K concentrations and resorption but not their utilization in a desert phreatophyte in its hyper-arid environment. This study provides a new insight into the phreatophytic plant nutrient cycle strategy under a changing external environment in a hyper-arid ecosystem.

## Introduction

Nutrient utilization and resorption are vital for plant growth and ecosystem processes in various ecosystems (Aubrey et al., 2012; Zhao et al., 2017; Wang et al., 2020). First, the total net primary production per unit nutrient absorption is described as nutrient use efficiency (NUE) (Vitousek, 1982). NUE is defined as the product of the mean residence time (MRT) of nutrients in plants and nutrient productivity (NP), which is the rate of biomass increase per unit nutrient in plants (Berendse and Aerts, 1987); a functional interpretation of NUE is considered in accordance with different nutrient use strategies: high NP is strongly associated with low MRT and NP is not related to MRT (Eckstein and Karlsson, 2001; Yuan et al., 2008). Previous studies showed that NUE is affected by nutrient availability and species. For example, NUE decreases as nutrient availability increases (Vitousek, 1982). The effect of species on NUE remains unclear, with one report suggesting that the NUE of C4 grass (*Chloris virgata*) is higher than that of the C3 grass (*Leymus chinensis*) despite nitrogen treatments in the temperate grasslands in northern China (Yuan et al., 2007), and the NUE of tree species is influenced by species but not by N availability (Aubrey et al., 2012). Other suggested that the NUE of woody shrubs and perennial herbs are similar among species and different habitats (high and low water supply) in a typical agropastoral ecotone in the central part of Inner Mongolia Autonomous Region, China (Yuan et al., 2007). Thus, knowledge gap on the roles of species type and nutrient availability in affecting NUE still exists in various ecosystems.

Nutrient resorption from senescing tissues to green tissues has important consequences for plants that live in nutrient-limited habitats. Nutrient resorption helps plants re-use nutrients directly to become less dependent on external nutrient concentrations (Aerts and Chapin, 2000; Wang et al., 2014, 2020; Zhao et al., 2017; Huang et al., 2018). Hence, nutrient resorption is an important mechanism of nutrient conservation in plants. Nutrient resorption efficiency (NRE) is a crucial indicator of internal nutrient recycling in plants (Aerts, 1996; Killingbeck, 1996). The NRE of plants is the percentage of nitrogen (N,P, and K) removed from senescing tissues. On a global scale, the mean N and P resorption efficiencies from senescent to young leaves of terrestrial plants are approximately 62 and 65%, respectively (Vergutz et al., 2012). NRE is usually higher in nutrient-limited habitats than in nutrient-rich ones (Killingbeck, 1996; Yuan and Chen, 2009). Recently, evidence has suggested that NRE is affected by climate, soil/leaf nutrient and stoichiometry, plant functional group, plant age, spatial pattern, and land utilization (Brant and Chen, 2015; Netzer et al., 2017; Xu et al., 2017; Zhao et al., 2017; Wang et al., 2020). Previous studies have shown that increasing water condition decreases the NRE of N, but increases that of P of three graminoid species, *Agropyron cristatum*, *Achnatherum sibiricum*, and *Stipa grandis*, and one forb species, *Potentilla bifurca* in Inner Mongolia, China (Lü and Han, 2010). Huang et al. (2018) reported that the NRE of N and P of three life-form plants (five spring annuals, two summer annuals and two shrubs) are not influenced by increasing water condition in a temperate desert in China. Hence, it is unclear if changing water condition affects NRE of plants in natural environment.

Phreatophytes are largely distributed worldwide except in Antarctica. The deep roots of phreatophytic plants depend on access to groundwater as a major source of nutrients and water (Arndt et al., 2004; Zeng et al., 2006; Thomas, 2014). However, with intensive human intervention, increasing anthropogenic water use intensifies groundwater limitation (Antunes et al., 2018). Groundwater table is gradually lowered in desert oasis transition ecotones, indicating the increasing shortage of water resources (Liu, 2007). Thus, limited plant access to groundwater caused by groundwater lowering is expected to have a major impact on plant nutrient utilization and absorption. Therefore, nutrient utilization and absorption of phreatophytes in nutrient-deficient deserts in response to the declining groundwater depth should be investigated to restore sparse phreatophytes and enhance protection for fragile arid desert ecosystems.

The Taklimakan Desert is the second largest shifting desert in the world and the largest desert in China. As a dominant perennial phreatophyte grown in arid and semi-arid regions between oasis and deserts, *Alhagi sparsifolia* Shap. can stabilize sand dunes, prevent land erosion, and thus support fragile desert ecosystems (Zeng and Liu, 2012; Li et al., 2015; Zhang et al., 2018a,b). *A. sparsifolia* is a leguminous plant that can fix atmospheric N_2_ (Arndt et al., 2004). It is also an economically important species, which can be used as a livestock source by local farmers in winter (Li et al., 2013; Zhang et al., 2018b). The deep root system of *A. sparsifolia* can reach groundwater, which is the major source of water and nutrients (Arndt et al., 2004; Zeng et al., 2006). Our previous study found that the declining water table affects the biomass, leaf physiological parameters, leaf nutrient, root characteristics, clonal growth, and propagation traits of *A. sparsifolia* (Gui et al., 2013; Zeng et al., 2013; Li et al., 2015; Zhang et al., 2018a).

Although changes in the NP, MRT, and NUE of nitrogen (N) have been widely explored, variations in the NUE of phosphorus (P) and potassium (K) in plants have been seldom investigated. Most studies have assessed nutrient resorption in woody, deciduous, and evergreen plants in response to environmental factors. However, only a few ones have investigated N, P, and K utilization and resorption in desert phreatophytes with a deep root system in a reduced groundwater depth in a hyper-arid region. In the present study, we hypothesize that varying groundwater depth can affect variations of NUE and NRE of N, P, and K in *A. sparsifolia* because of the changes in N, P, and K concentrations in *A. sparsifolia* in a hyper-arid desert ecosystem. Desert phreatophytes, such as *A. sparsifolia*, can adjust NUE and NRE to adapt to declining groundwater depth in a hyper-arid desert ecosystem.

## Materials and Methods

### Study Area

The study site is located at the Cele National Station of Observation and Research for Desert and Grassland Ecosystem (37°00.77″N, 80°43.45″E, Supplementary Figure 1) at the southern edge of the Taklimakan Desert in the Xinjiang Uyghur Autonomous Region of China. Cele Station located in the desert-oasis transition ecotone is characterized by a 5–10 km belt of sparse phreatophytic species dominated by *A. sparsifolia* (Zeng et al., 2013). The groundwater depth ranges from 2.5 to 15.0 m in this area. The study site has an elevation of 1366 m (asl), a mean annual temperature of 11.9°C, a mean annual potential evaporation of 2600 mm, and a mean annual precipitation of 35 mm (Gui et al., 2013; Liu et al., 2016). The climate is hyper-arid, with hot and dry summers and cold and dry winters. Extreme temperatures can reach 41.9°C in summer and −31°C in winter. Soils are sandy, and their bulk density is 1.35 g/cm^3^ (Liu et al., 2013, 2016). Soil C, N, and P concentrations at 2.5 m groundwater depth are similar to those at 4.5 m groundwater depth, while soil C, N, and P concentrations at 2.5 and 4.5 m groundwater depths are significantly lower than those at 11.0 groundwater depth (Zhang et al., 2018a).

Three sites with varying groundwater depths were selected: 2.5 m (37°01′18″N, 80°42’29″E), 4.5 m (37°00′40″N, 80°42′13″E), and 11.0 m (37°00′33″N, 80°42′25″E, Supplementary Figure 2). In each research area, the groundwater depth in a well was measured every month. The fluctuation in groundwater depth was minor from April to October in 2015 and 2016. Each site has a large area of approximately 2 ha. At each sampling stage, three plots with an area of 9 m^2^ were randomly selected at each site.

### Field Sampling and Chemical Analysis

At each groundwater depth, three *A. sparsifolia* plants in different plots with similar canopy sizes, heights, and numbers of stems were selected each month from June to October 2015 and 2016. In the experimental period, nine plots were sampled nine times per month. Thus, 45 samplings in 2015 and 2016 were performed. During sampling, plant above-ground was collected by chipping at the ground level and further separating into different tissues, including leaf, stem, and assimilating branch. The samples were oven-dried at 105°C for 15 min and at 70°C to a constant weight. The dry biomass of leaf, stem, and the assimilating branches of *A. sparsifolia* were weighed. All samples were subsequently ground using a miller and sieved through a 1 mm mesh screen for chemical element analysis. Plant tissues were digested using 0.02 M sulfuric acid, and N concentrations were determined by the Kjeldahl acid-digestion method (Sparks et al., 1996). The P and K concentrations were determined using the colorimetric analysis and a flame analyzer (2655–00, Chicago, United States), respectively, after digestion with sulfuric acid (Thomas et al., 1967).

### Definition and Calculations

NUE (g g^–1^) is calculated as the product of NP (g g^–1^ year^–1^) and MRT (year) of nutrients (Berendse and Aerts, 1987; Garnier and Aronson, 1998; Yasumura et al., 2002; Yuan et al., 2004, 2006, 2007; Norby and Iversen, 2006). NP was calculated for short intervals (Eckstein and Karlsson, 2001; Yuan et al., 2004, 2007) in accordance with the following equation:

$$\text{NP}=\frac{M_{2}-M_{1}}{t_{2}-t_{1}}\cdot \frac{l\,n\,N_{2}-l\,n\,N_{1}}{N_{2}-N_{1}}$$

where *M* is biomass of *A. sparsifolia* and *N* is the average leaf, stem, and assimilating branch N concentration, respectively, in two consecutive harvests conducted at times *t*_1_ and *t*_2_. The mean annual nutrient concentration was estimated as a weighted average in the entire year of 2015 and 2016. Besides, MRT was calculated as the ratio between the average nutrient concentration of leaf, stem, and assimilating branch of *A. sparsifolia* and the annual nutrient losses of *A. sparsifolia* (Eckstein and Karlsson, 2001; Norby and Iversen, 2006). Nutrient losses were determined on the nutrient concentration of the aboveground dead *A. sparsifolia* harvested in October 2015 and 2016.

Nutrient concentrations in green and senescing tissues of *A. sparsifolia* were used to calculate N-resorption efficiency (NRE, %) = [(Ng–Ns)/Ng]⋅100, where Ng and Ns are the nutrient concentrations in green and senescing tissues of *A. sparsifolia*, respectively. Nutrient concentration in senescing tissues of *A. sparsifolia* (October 2015 and 2016) was used as a direct indicator of nutrient resorption proficiency (NRP), that is, the extent to which nutrient concentration is reduced in senescent tissues. A high resorption proficiency usually indicates low nutrient concentration in senesced tissues (Killingbeck, 1996; Richardson et al., 2005; Yuan et al., 2007; Wang et al., 2014).

### Data Analysis

One-way ANOVA with Tukey’s test was carried out for the multiple comparisons of N, P, and K concentrations, NP, MRT, NUE, NRE, and NRP at different groundwater depths to test statistical significance (*p* < 0.05). Analyses were performed using SPSS 19.0. Piecewise structural equation modeling (piecewise SEM) was used to statistically tease apart and quantify direct and indirect effects of groundwater depth on plant leaf nutrient, nutrient utilization and resorption using R software (version 4.0.3) with the package “piecewiseSEM” (Lefcheck, 2016). D-separation test of Piecewise SEM was used to evaluate whether the causal model misses important links and *p* > 0.05 indicates that the model is acceptable (Shipley, 2002).

## Results

### N, P, and K Concentrations of *A. sparsifolia* as Affected by Groundwater Depth

**FIGURE 1:**
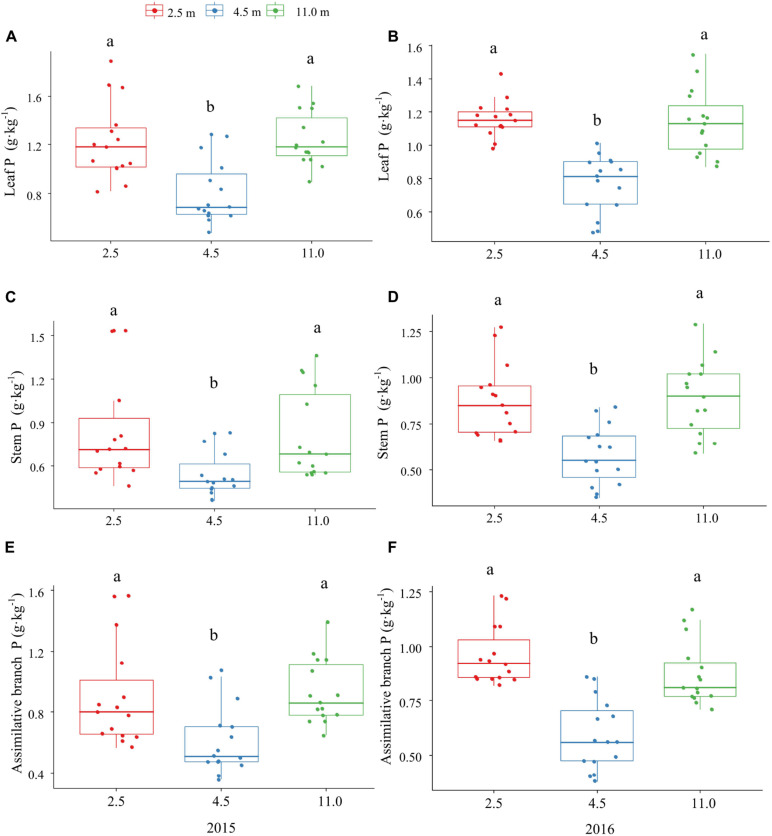
Changes in the P concentrations in leaf, stem, and assimilating branch of *A. sparsifolia* at 2.5, 4.5, and 11.0 m groundwater depths in the growing season in 2015 and 2016. The vertical bars denote standard deviations and letters indicate significant differences among the different groundwater depths (*p* < 0.05). **(A,C,E)** represent variations of leaf P, stem P, and assimilative branch P under different groundwater depths in 2015. **(B,D,F)** represent variations of leaf P, stem P, and assimilative branch P under different groundwater depths in 2016.

**FIGURE 2:**
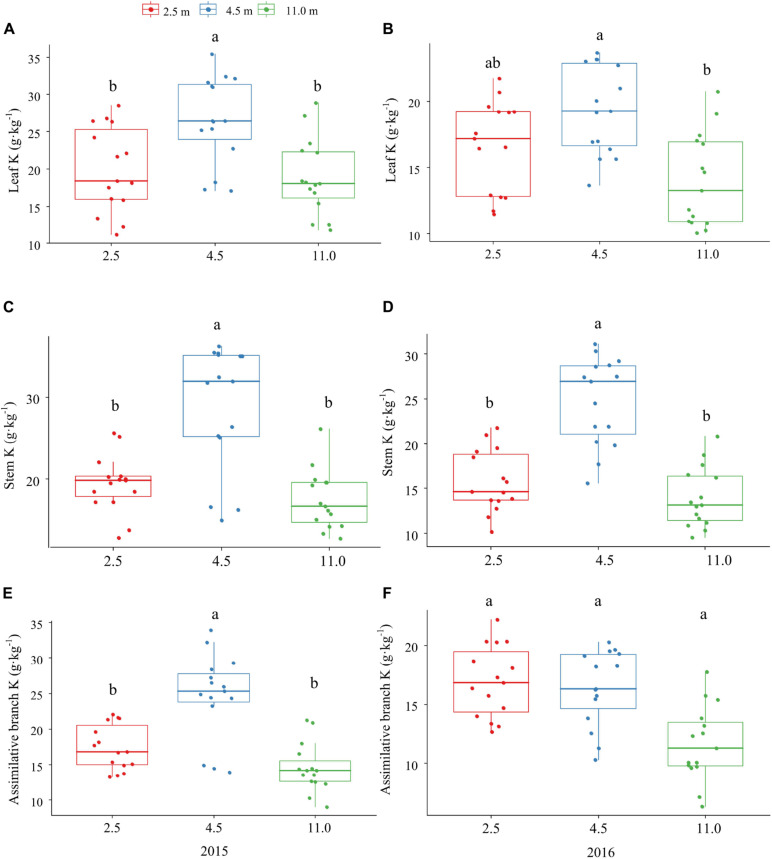
Changes in the *K* concentrations in leaf, stem, and assimilating branch of *A. sparsifolia* at 2.5, 4.5, and 11.0 m groundwater depths in the growing season in 2015 and 2016. The vertical bars denote standard deviations and letters indicate significant differences among the different groundwater depths (*p* < 0.05). **(A,C,E)** represent variations of leaf K, stem K, and assimilative branch K under different groundwater depths in 2015. **(B,D,F)** represent variations of leaf K, stem K, and assimilative branch K under different groundwater depths in 2016.

**FIGURE 3:**
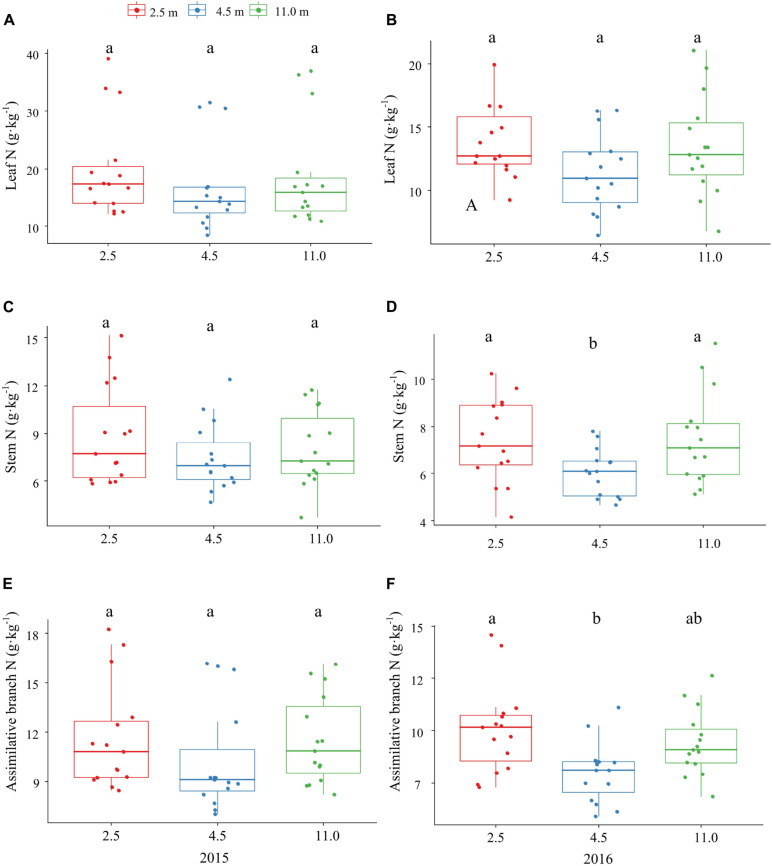
Changes in the N concentrations in leaf, stem, and assimilating branch of *A. sparsifolia* at 2.5, 4.5, and 11.0 m groundwater depths in the growing season in 2015 and 2016. The vertical bars denote standard deviations and letters indicate significant differences among the different groundwater depths (*p* < 0.05). **(A,C,E)** represent variations of leaf N, stem N, and assimilative branch N under different groundwater depths in 2015. **(B,D,F)** represent variations of leaf N, stem N, and assimilative branch N under different groundwater depths in 2016.

The P and K concentrations in the leaf, stem, and assimilating branch of *A. sparsifolia* were significantly affected by groundwater depth (Figures 1, 2). The P concentration in different tissues of *A. sparsifolia* at 4.5 m groundwater were significantly lower than those at 2.5 and 11.0 m groundwater depths (Figure 1). The K concentration in all three parts of *A. sparsifolia* at 4.5 m groundwater depth were the highest among the three groundwater depths. The K concentration in all parts of *A. sparsifolia* at 2.5 m groundwater depth were greater than those at 11.0 m groundwater depth (Figure 2). However, the N concentration in leaf, stem, and assimilating branch of *A. sparsifolia* were not influenced by groundwater depth in both years (Figure 3).

### N, P, and K Use and Resorption Strategies as Affected by Groundwater Depth

Groundwater depth significantly influenced the P resorption parameters, such as NRE and NRP of P but not the P utilization parameters, such as NP, MRT, and NUE of P (Table 1). The NRE of P was significantly greater at 4.5 m groundwater depth than at 2.5 and 11.0 m groundwater depths in 2015 and 2016, corresponding to the lower NRP of P at 4.5 m groundwater depth than at 2.5 and 11.0 m groundwater depths in 2015 and 2016, respectively. Groundwater depth did not affect the N utilization and resorption parameters, such as NP, MRT, NUE, NRE, and NRP of N in *A. sparsifolia* in both years (Table 2).

**TABLE 1:** Phosphorus use strategies of *A. sparsifolia* affected by 2.5, 4.5, and 11.0 m groundwater depths in 2015 and 2016.

2015	NP (g g^–1^ year^–1^)	MRT (year)	NUE (g g^–1^)	NRE (%)	NRP (mg g^–1^)
2.5 m	8.77 ± 3.10^a^	1.36 ± 0.40^a^	12.68 ± 6.79^a^	49.08 ± 9.76^ab^	0.83 ± 0.15^a^
4.5 m	4.55 ± 2.72^a^	0.97 ± 0.05^a^	4.46 ± 2.81^a^	50.15 ± 10.37^a^	0.49 ± 0.08^*b*^
11.0 m	3.86 ± 3.26^a^	0.95 ± 0.08^a^	3.83 ± 2.81^a^	29.15 ± 2.71^*b*^	0.87 ± 0.08^a^
2016	NP (g g^–1^ year^–1^)	MRT (year)	NUE (g g^–1^)	NRE (%)	NRP (mg g^–1^)
2.5 m	4.38 ± 1.14^a^	1.51 ± 0.19^a^	6.48 ± 1.23^a^	69.74 ± 4.75^*b*^	0.83 ± 0.11^a^
4.5 m	4.58 ± 2.46^a^	1.74 ± 0.54^a^	7.46 ± 3.37^a^	81.07 ± 4.32^a^	0.42 ± 0.01^*b*^
11.0 m	2.73 ± 0.59^a^	2.19 ± 0.40^a^	5.91 ± 1.36^a^	71.81 ± 2.78^ab^	0.84 ± 0.07^a^

*Means followed by different letters are significantly different at *p* < 0.05.*

*NP, nutrient productivity; MRT, mean residence time; NUE, nutrient-use efficiency; NRE, nutrient-resorption efficiency; NRP, nutrient-resorption proficiency.*

*“ab” means that there are no significant differences among a, ab, and b at *p* < 0.05.*

**TABLE 2:** Nitrogen use strategies of *A. sparsifolia* affected by 2.5, 4.5, and 11.0 m groundwater depths in 2015 and 2016.

2015	NP (g g^–1^ year^–1^)	MRT (year)	NUE (g g^–1^)	NRE (%)	NRP (mg g^–1^)
2.5 m	0.62 ± 0.20^a^	1.33 ± 0.32^a^	0.86 ± 0.40^a^	46.25 ± 6.20^a^	11.76 ± 1.35^a^
4.5 m	0.26 ± 0.16^a^	0.94 ± 0.05^a^	0.24 ± 0.16^a^	55.14 ± 4.11^a^	8.43 ± 0.56^a^
11.0 m	0.40 ± 0.05^a^	0.95 ± 0.07^a^	0.38 ± 0.07^a^	46.16 ± 8.29^a^	11.08 ± 2.03^a^
2016	NP (g g^–1^ year^–1^)	MRT (year)	NUE (g g^–1^)	NRE (%)	NRP (mg g^–1^)
2.5 m	0.52 ± 0.16^a^	1.14 ± 0.05^a^	0.59 ± 0.17^a^	47.34 ± 7.44^a^	9.37 ± 1.49^a^
4.5 m	0.42 ± 0.22^a^	1.04 ± 0.15^a^	0.43 ± 0.18^a^	52.90 ± 8.69^a^	7.29 ± 0.30^a^
11.0 m	0.34 ± 0.09^a^	1.74 ± 0.59^a^	0.60 ± 0.31^a^	50.58 ± 5.31^a^	9.23 ± 1.37^a^

*Means followed by different letters are significantly different at *p* < 0.05.*

*NP, nutrient productivity; MRT, mean residence time; NUE, nutrient-use efficiency; NRE, nutrient-resorption efficiency; NRP, nutrient-resorption proficiency.*

*“a” means that there is no significant difference in the three groundwater depths.*

Similarly, groundwater depth significantly affected the K resorption parameters, such as NRE and NRP of K, but not the K utilization parameters, such as NP, MRT, and NUE (Table 3). The NRE of K at 11.0 m groundwater depth was the greatest among the three groundwater depths, and the NRP at 11.0 m groundwater depth was the lowest in 2015. The NRE of K at 4.5 m groundwater depth was the lower than that at 2.5 and 11.0 m groundwater depth, corresponding to the highest NRP at 4.5 m groundwater depth in 2015.

**TABLE 3:** Potassium use strategies of *A. sparsifolia* affected by 2.5, 4.5, and 11.0 m groundwater depths in 2015 and 2016.

2015	NP (g g^–1^ year^–1^)	MRT (year)	NUE (g g^–1^)	NRE (%)	NRP (mg g^–1^)
2.5 m	7.30 ± 4.17^a^	0.98 ± 0.29^a^	7.93 ± 5.47^a^	17.92 ± 2.14^ab^	16.78 ± 1.12^*b*^
4.5 m	4.21 ± 2.91^a^	0.76 ± 0.11^a^	3.15 ± 2.36^a^	7.21 ± 4.95^*b*^	31.62 ± 1.29^a^
11.0 m	4.04 ± 2.44^a^	0.65 ± 0.08^a^	2.67 ± 1.38^a^	25.49 ± 5.91^a^	12.11 ± 0.42^*c*^
2016	NP (g g^–1^ year^–1^)	MRT (year)	NUE (g g^–1^)	NRE (%)	NRP (mg g^–1^)
2.5 m	0.95 ± 0.47^a^	0.85 ± 0.15^a^	0.76 ± 0.31^a^	13.66 ± 6.33^a^	17.62 ± 0.72^a^
4.5 m	1.01 ± 0.52^a^	0.70 ± 0.04^a^	0.71 ± 0.38^a^	19.96 ± 1.07^a^	20.33 ± 3.09^a^
11.0 m	0.41 ± 0.27^a^	1.30 ± 0.51^a^	0.47 ± 0.28^a^	17.41 ± 9.12^a^	14.64 ± 4.58^a^

*Means followed by different letters are significantly different at *p* < 0.05.*

*NP, nutrient productivity; MRT, mean residence time; NUE, nutrient-use efficiency; NRE, nutrient-resorption efficiency; NRP, nutrient-resorption proficiency.*

### Factors Affecting Variation in N, P, and K Resorption

**FIGURE 4:**
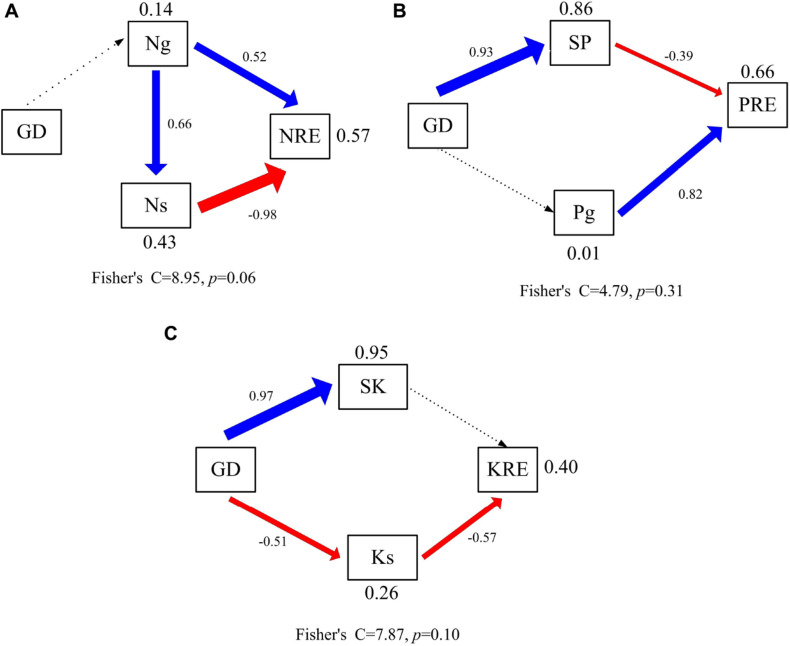
Controlling factors analysis of leaf resorption efficiency using the structural equation model. Significant regressions are indicated by solid lines (*p* < 0.05) and nonsignificant regressions by dashed lines. The thickness of the significant paths has been scaled based on the magnitude of the standardized regression coefficient. Black arrows denote positive relationships, and red arrows negatives ones. GD, groundwater depth; Ng, nitrogen concentrations of green leaves; Ns, nitrogen concentrations of senesced leaves; NRE, nitrogen resorption efficiency; SP, soil available phosphorus concentration; Pg, phosphorus concentrations of green leaves; PRE, phosphorus resorption efficiency; SP, soil available potassium concentration; Ks, potassium concentrations of senesced leaves; KRE, potassium resorption efficiency. **(A)** Controlling factors analysis of leaf nitrogen resorption efficiency. **(B)** Controlling factors analysis of leaf phosphorus resorption efficiency. **(C)** Controlling factors analysis of leaf potassium resorption efficiency.

The factors affecting variation in N, P, and K resorption were explored using structural equation models (SEM) (Figure 4). The model explained 57, 66, and 40% of the variance in NRE of N, P, and K (Figures 4A–C). Groundwater depth had no direct and indirect effects on the NRE of N (Figure 4A). The NRE of N was directly affected by green leaf and senesced leaf N concentrations. In addition, groundwater depth had indirect effect on the NRE of P (Figure 4B). The NRE of P was directly affected by green leaf P concentrations and soil P concentration. Soil P concentration was most positively related to groundwater depth. Moreover, groundwater depth had an indirect effect on the NRE of K (Figure 4C). The NRE of K was directly affected by senesced leaf K concentration. Besides, senesced leaf K concentration was negatively related to groundwater depth.

## Discussion

### Effect of Groundwater Depth on N, P, and K Concentrations of *A. sparsifolia*

Our results showed that groundwater depth had a significant effect on P concentrations in different tissues of phreatophytic *A. sparsifolia.* This result is consistent with that of previous studies that leaf P concentration of *A. sparsifolia* was significantly affected by groundwater depth. This is consistent with the observation that leaf P concentration of *A. sparsifolia* at 4.5 m is lower than those at 2.5 and 11.0 m groundwater depths in 2015 and 2016 because biomass of *A. sparsifolia* at 4.5 m is geater than those at 2.5 and 11.0 m groundwater depths. Hence, a biomass dilution effect existed in leaf P concentration of *A. sparsifolia* at 4.5 m groundwater depths (Zhang et al., 2018a). Moreover, the effect of groundwater depth on leaf P concentration is likely caused by direct effect of groundwater on soil P concentration (Figure 4B), which is strongly related to the plant P concentration of *A. sparsifolia* (Zhang et al., 2018a). These results are in accordance with previous experiments that soil P concentration is the key factor affecting the growth and salt Tolerance of *A. sparsifolia* in different habitat in Northwest China (Zhang et al., 2018b).

In addition, K concentration in phreatophytic *A. sparsifolia* was significantly affected by groundwater depth. The leaf, stem, and assimilating branch K concentrations of *A. sparsifolia* at 4.5 m groundwater depth were significantly higher than those at 2.5 and 11.0 m groundwater depths and is mainly due to the direct negative effect of groundwater depth on senesced leaf K concentration (Figure 4C) and leaf K concentration is affected by soil K concentration (Zeng et al., 2001) and water stress (Saneoka et al., 2004). Moreover, N concentration in the phreatophytic *A. sparsifolia* was unaffected by various groundwater depth because this plant is a leguminous plant, and more than 80% of its total leaf N is fixed by the atmosphere (Arndt et al., 2004).

### Effect of Groundwater Depth on the Nutrient Resorption of *A. sparsifolia*

Herein, our data partly supported the hypothesis that groundwater depth had a significant influence on the nutrient resorption (NRE of P and K) of the phreatophytic *A. sparsifolia*, but its effect on nutrient utilization (NUE of N, P, and K) was minimal. Groundwater depth significantly affected the NRE of P in *A. sparsifolia*. Although groundwater depth had no direct effect of the NRE of P, it had an indirect effect on the NRE of P via soil P concentration that is mostly determined by groundwater depth (Figure 4). This result is similar to that of our previous experiments that leaf P concentration in *A. sparsifolia* is positively linked to soil P concentration under different groundwater depths (Zhang et al., 2018a). Similar reports have noted that the NRE of P in herbaceous species decreases with increasing soil P concentration as indicated by global data analysis (Wang et al., 2018).

The NRE of P in *Cleistogenes squarrosa* (Trin. ex Ledeb.) Keng is significantly affected by green and senesced leaf P concentrations in overgrazing treatments (Wang et al., 2020). Herein, the NRE of P in *A. sparsifolia* at 4.5 m groundwater depth was significantly higher than that at 2.5 m (Table 1), suggesting that *A. sparsifolia* at 4.5 m groundwater depth could have translocated more P from the senescing tissues to the green tissues to support plant growth as indicated in our previous report that the soil P concentrations between 4.5 and 2.5 m groundwater depths in the same area were not significantly different (Zhang et al., 2018a). This funding is also supported by the lower P concentration in senescent tissues (NRP of P) at 4.5 m groundwater depth than that at 2.5 m (Table 1 and Figure 1). The NRE of P in *A. sparsifolia* at 4.5 m groundwater depth was significantly greater than that at 11.0 m groundwater depth because soil available P concentration at 4.5 m groundwater depth is significantly lower than those at 11.0 m groundwater depth (Zhang et al., 2018a). The results suggested that *A. sparsifolia* in the P-limited habitat adopt better nutrient conservation mechanisms to reduce nutrient loss through internal nutrient cycling. This result is also consistent with the observation that desert plants in nutrient-poor environments usually rely on high NRE to alleviate nutrient limitation (Drenovsky and Richards, 2006; Vergutz et al., 2012; Brant and Chen, 2015).

In the present study, the observed NRE of P in *A. sparsifolia* under different groundwater depths ranges from 29.2 to 81.1% and it similar to that of a legume (*Oxytropis* sp.) that grows on the Tibetan Changtang Plateau (Zhao et al., 2017). The P resorption efficiency in 2015 was 29.15–50.15%, which was lower than the global value of 64.9%; the P resorption efficiency values in 2016 was 69.74–81.07% and greater than the global P resorption efficiency (Vergutz et al., 2012). There are significant difference between NRE of P in *A. sparsifolia* in 2015 and 2016 and interactive effects of groundwater depth and year were found on NRE of P in *A. sparsifolia* in 2015 and 2016 (Supplementary Table 2). It is important to note that leaf P resorption enhances with increasing mean annual precipitation (Brant and Chen, 2015). Leaf P resorption efficiencies of woody plants increase with mean annual precipitation at the global scale (Yuan and Chen, 2009), and P resorption efficiencies of a legume (*Oxytropis* sp.), grass (*S. purpurea*), and forb (*Potentilla bifurca* L) are enhanced with increasing precipitation on the Tibetan Changtang Plateau (Zhao et al., 2017). Therefore, the significant difference between NRE of P in *A. sparsifolia* in 2015 and 2016 was likely due to the limited precipitation in 2015 (34.2 mm), which was 26.9% less than that in 2016 (43.4 mm, Supplementary Table 1). These results are in accordance with previous findings of Ren et al. (2018) who reported that the nutrient resorption of two perennial grasses, *Cleistogenes songorica*, and *Stipa breviflora*, and one perennial semi-shrub, *Artemisia frigida*, to warming and nitrogen fertilization could be regulated by natural precipitation variations in a desert grassland in Inner Mongolia, northern China. These results are also consistent with the observation that leaf P resorption in Lucerne enhances with increasing water conditions in the greenhouse (Lu et al., 2019).

Similarly, groundwater depth had no direct effect on the NRE of K, had an indirect effect on the NRE of K via the senesced leaf K concentration (Figure 4). The NRE of K in *A. sparsifolia* at 11.0 m groundwater depth was the higher than 2.5 and 4.5 m groundwater depths, while the NRP of K in *A. sparsifolia* at 11.0 m groundwater depth was the lower than the 2.5 and 4.5 m groundwater depths in 2015 (Table 3). As such, *A. sparsifolia* at 11.0 m groundwater depth had a greater NRE of K than others, resulting in the lowest K concentration in senescent tissues (NRP of K) to adapt to the largest groundwater depth at the southern rim of the Taklimakan Desert (Figure 2). The NRE of K in *A. sparsifolia* under different groundwater depths ranged from 7.2 to 25.5% in 2015 and 13.7 to 20.0% in 2016, respectiely, which was significantly lower than the highest global K resorption efficiency of 70.1% (Vergutz et al., 2012) and in lucerne that varies from 21.0 to 49.8 % with an average of 36.9 % (Wang et al., 2014). Besides, no significant difference between NRE of K was observed in *A. sparsifolia* in 2015 and 2016 (Supplementary Table 2). These results may because sand soil in the desert had higher K concentration and lower soil N and P concentrations (Liu et al., 2013), and the NRE in nutrient-rich habitats is lower than that in nutrient-poor habitats, compared with other soil types (Killingbeck, 1996; Yuan and Chen, 2009). Hence, the low NRE of K in *A. sparsifolia* was attributed to the high K availability in soil and the high K availability in soil results in no significant difference between NRE of K in *A. sparsifolia* in 2015 and 2016. In the present study, groundwater depth had no effect on the NRE of N (Figure 4). The NRE of N in *A. sparsifolia* under different groundwater depths was 46.1–55.1% and significantly lower than the highest global N resorption efficiency (62.1%) (Vergutz et al., 2012) and the NRE in grass (*Stipa purpurea*; 74.4%), sedge (*Carex moorcroftii*; 73.7%), and forb (*Potentilla bifurca* L; 73.5%) (Zhao et al., 2017). However, the observed values were similar to that of a legume (*Oxytropis* sp.) on the Tibetan Changtang Plateau (47.2%) (Zhao et al., 2017) and higher than that of lucerne (a perennial legume) with an average of 16.2% (Wang et al., 2014). These results suggested that *A. sparsifolia* can fix atmospheric N_2_, resulting in a relatively lower resorption efficiency of N in *A. sparsifolia* than in non-legume plants.

### Effect of Groundwater Depth on Nutrient Utilization in *A. sparsifolia*

In the present study, the NUE of P and K were decreased with increasing groundwater depth, while the NUE of P and K among 2.5, 4.5, and 11.0 m groundwater depth had no significant differences (Tables 1–3). This result is mainly due to *A. sparsifolia* that adapt to the changes only by adjusting the nutrient resorption process rather than the nutrient utilization process. This result is similar to those of previous studies that the NUE of six species were similar at high and low water supply treatments in a typical agropastoral ecotone in China (Yuan et al., 2008), and the NUE of tree species depended on the species but not on nutrient availability (Aubrey et al., 2012). The NUE of N at 4.5 m groundwater depth was lower than that of 2.5 and 11.0 m groundwater depths because *A. sparsifolia* is a leguminous plant that can fix N in air, its biomass at 4.5 m groundwater depth is higher than that at 2.5 and 11.0 m groundwater depths (Zhang et al., 2018a). Future research should focus on the effects of nutrient and salinity in groundwater combined with groundwater depth on the nutrient resorption of phreatophytes. Long-term data on plant and soil in control and field experiments are also needed to measure and understand the responses of phreatophytes to groundwater drawdown combined with global change, such as variations in precipitation, temperature, and nitrogen deposition. In addition, only one phreatophyte chooses in this study, so if other phreatophytes have similar nutrient cycling strategies will need more research work in the future.

## Conclusion

Groundwater depth significantly affected P and K concentrations in leaf, stem, and assimilating branch of *A. sparsifolia*, but did not influence N concentrations in different tissues of *A. sparsifolia* in the southern rim of the Taklimakan Desert. *A. sparsifolia* under different groundwater depths drastically altered the nutrient resorption parameters, such as NRP and NRE of P and K, but not the nutrient uptake parameters, such as NP, MRT, and NUE. Besides, groundwater depth had significant indirect effects on the P and K resorption of *A. sparsifolia* by changing soil P concentration and senescent leaf K concentration. Hence, the variations in P and K concentrations in *A. sparsifolia* under different groundwater depths were highly associated with their resorption rather than their utilization by plants. Our results presented a new internal nutrient cycling strategy for nutrient conservation of a desert phreatophyte grown under poor soil fertility at different groundwater depths in a desert-oasis ecotone. This study may contribute to protecting and restoring phreatophytes in a hyper-arid desert ecosystem.

## Data Availability Statement

The original contributions presented in the study are included in the article/Supplementary Material, further inquiries can be directed to the corresponding author/s.

## Author Contributions

BZ, XG, and FZ designed the study. BZ and SZ performed the experiments and collected the data. BZ analyzed the data. BZ, GT, HY, MS, BL, XG, and FZ interpreted the data and wrote the manuscript. All authors contributed to the article and approved the submitted version.

## Conflict of Interest

The authors declare that the research was conducted in the absence of any commercial or financial relationships that could be construed as a potential conflict of interest.
