# Supplementary material for: Groundwater Depths Affect Phosphorus and Potassium Resorption but Not Their Utilization in a Desert Phreatophyte in Its Hyper-Arid Environment
Source: Front Plant Sci. 2021 Jun 7;12:665168. doi: 10.3389/fpls.2021.665168 (PMC8216241; doi:10.3389/fpls.2021.665168)
Supplement: Supplementary file 1 [file Data_Sheet_1.docx]

**Supplementary materials**

Figure S1 The location of the study area.

Figure S2 The growth conditions of *A. sparsifolia* in the study site.


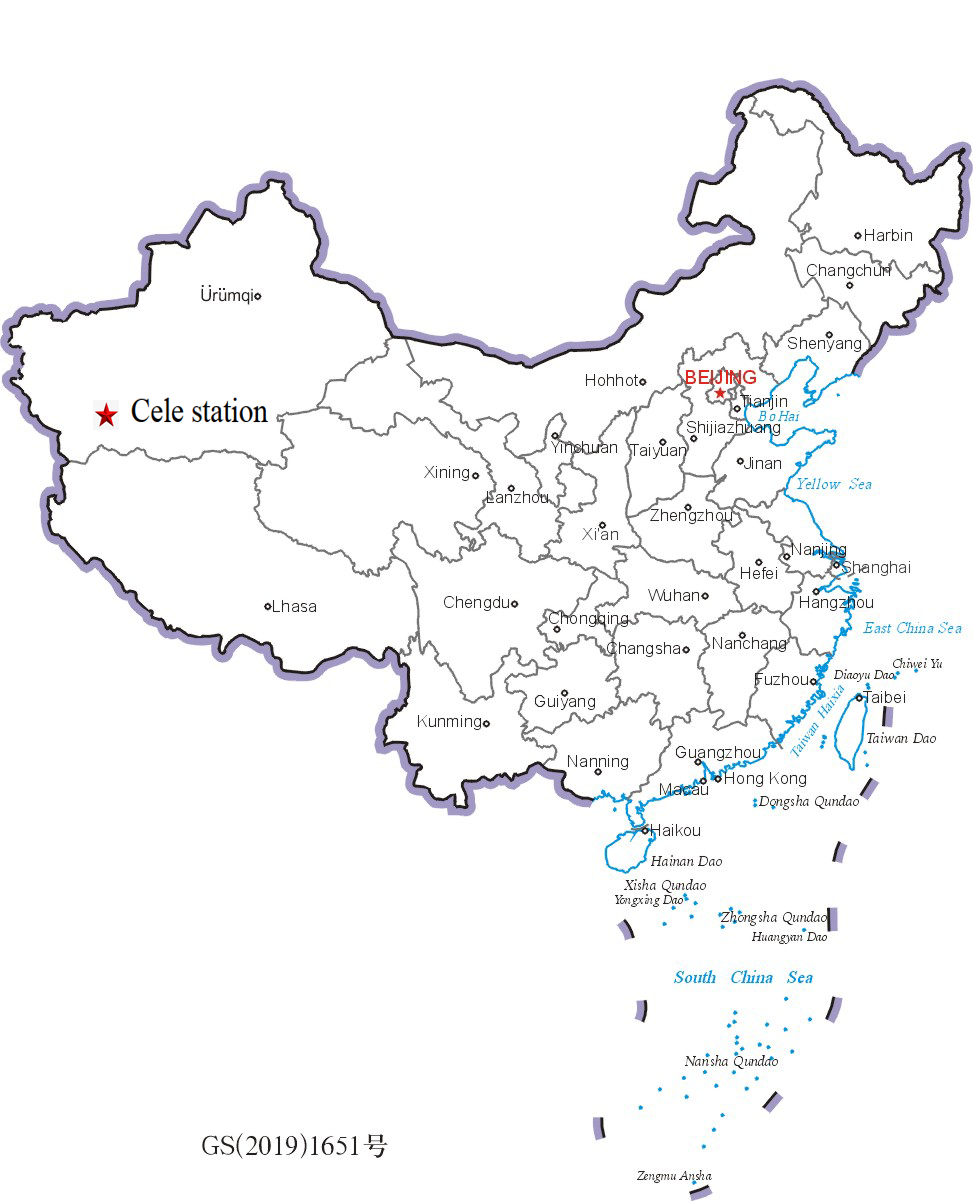


Fig. S1


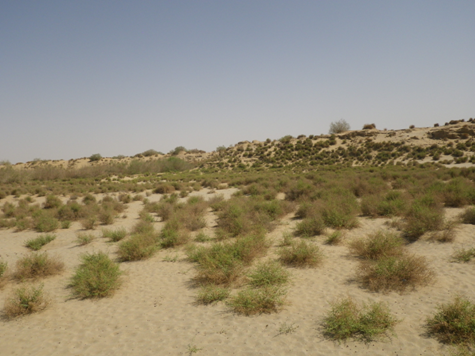


2.5 m groundwater depth


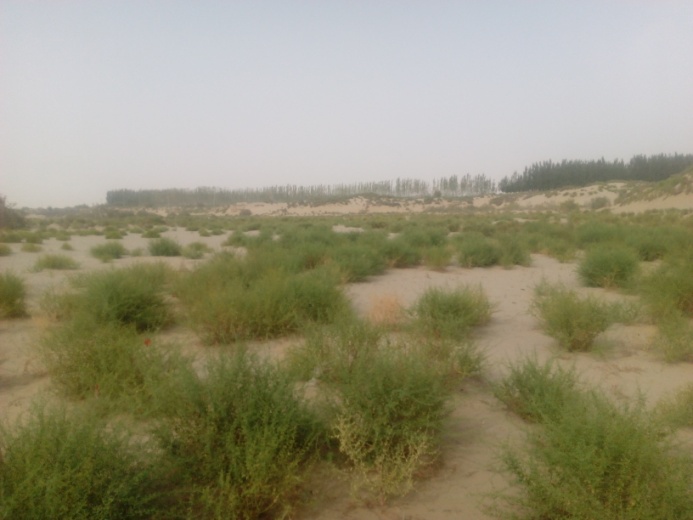

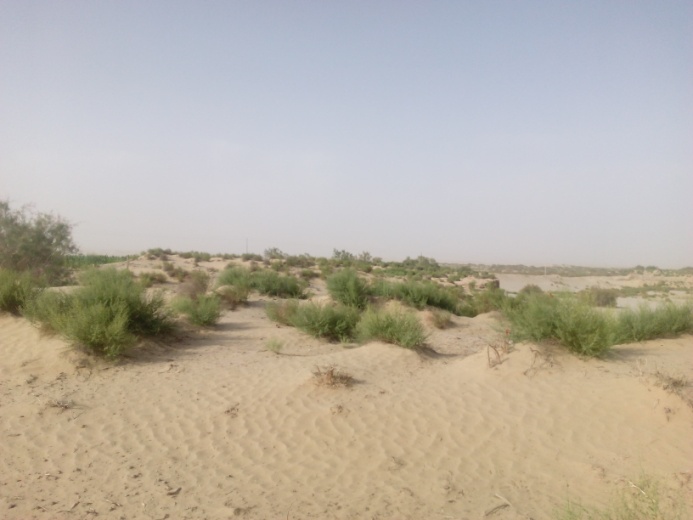


4.5 m groundwater depth

11.0 m groundwater depth

Fig. S2

Table S1 Precipitation of Cele Station in 2015 and 2016

| Year | May | June | July | Augest | September | October | Total |
| --- | --- | --- | --- | --- | --- | --- | --- |
| 2015 | 5.6 mm | 7.2 mm | 3.8 mm | **0.2 mm** | **17.4 mm** | 0.0 mm | 34.2 mm |
| 2016 | 3.4 mm | 1.8 mm | 1.2 mm | **15.2 mm** | **21.8 mm** | 0.0 mm | 43.4 mm |

Table S2 Results (P-values) of repeated measures ANOVA on the effects of groundwater depth (GD), year (Y), and their interactions on nitrogen resorption efficiency (NRE), phosphorus resorption efficiency (PRE), and potassium resorption efficiency (KRE).

|  | NRE | PRE | KRE |
| --- | --- | --- | --- |
| GD | 0.742 | **<0.001** | 0.958 |
| Y | 0.201 | **0.006** | 0.080 |
| GD×Y | 0.710 | **0.041** | **0.017** |
